# Myeloid‐Derived Suppressor Cells (MDSCs) Suppress T‐Cell Proliferation Less Than Mature Neutrophils in Blood and Bone Marrow From Multiple Myeloma Patients

**DOI:** 10.1155/jimr/9232540

**Published:** 2026-01-05

**Authors:** Julia Westerlund, Sandra Askman, Åsa Pettersson, Stina Wichert, Thomas Hellmark, Åsa C. M. Johansson, Markus Hansson

**Affiliations:** ^1^ Division of Hematology and Transfusion Medicine, Department of Laboratory Medicine, Lund University, BMC B13, Lund, 22184, Sweden, lu.se; ^2^ Department of Hematology, Oncology and Radiation Physics, Skåne University Hospital, Lund, 22185, Sweden, skane.se; ^3^ Department of Respiratory Medicine and Allergology, Skåne University Hospital, Lund, 22185, Sweden, skane.se; ^4^ Nephrology, Department of Clinical Sciences Lund, Skåne University Hospital, Lund University, Barngatan 2, Lund, 22185, Sweden, lu.se; ^5^ Clinical Genetics and Pathology, Skåne University Hospital, BMC C13, Lund, 22185, Region Skåne, Sweden, skane.se; ^6^ Department of Internal Medicine and Clinical Nutrition, University of Göteborg, Sahlgrenska Academy, Institute of Medicine, Bruna Stråket 5 Plan 5, Göteborg, 41325, Sweden, gu.se

## Abstract

Multiple myeloma (MM) is the second most common hematological malignancy, characterized by a clonal expansion of malignant plasma cells in bone marrow. Monoclonal gammopathy of undetermined significance (MGUS) is the premalignant condition of MM. The tumor microenvironment is thought to influence the progression from premalignant conditions. Myeloid‐derived suppressor cells (MDSCs) are a heterogenous group of different cellular subsets with myeloid origin, characterized by their ability to inhibit T‐cell responses. MDSC are thought to play an important immunoregulatory role in different diseases, and in many cancers their levels seem to correlate with a poor prognosis. There are three different subsets, the neutrophil‐like polymorphonuclear (PMN)‐MDSC, the monocyte‐like (M)‐MDSC, and the immature early (e)MDSC. In this study, we investigate the levels and functions of all MDSC subsets in the bone marrow of both MGUS and MM patients and compare it to blood MDSC. We found that MDSC levels are not increased in neither the blood nor bone marrow of MGUS or MM patients, and they lack strong T‐cell suppressive abilities. Blood PMN‐MDSC seems to have a small inhibitory effect, but mature neutrophils were more suppressive. Interestingly, eMDSC levels were decreased in the blood of MM patients. Our data indicate that MDSC are not key players in the pathogenesis of MM, but that mature neutrophils may be more important as they have a stronger immunoregulatory effect.

## 1. Introduction

Multiple myeloma (MM) is the second most common hematological malignancy and accounts for 1% of all cancer cases [1]. In MM, there is a clonal expansion of malignant plasma cells in the bone marrow that usually leads to increased levels of monoclonal immunoglobulins (M component) or light chains. MM is characterized by the CRAB criteria—increased calcium levels, renal failure, anemia, and osteolytic bone lesions [1]. Many MM patients also suffer from recurrent bacterial infections [2], which may suggest an impaired neutrophil function [3]. MM is preceded by a premalignant condition called monoclonal gammopathy of undetermined significance (MGUS) [4, 5]. MGUS is associated with a life‐long risk of developing MM and each year 1% of the patients with MGUS develop MM [4–6]. MGUS patients do not have any clinical symptoms of MM but have an overproduction of immunoglobulins from monoclonal plasma cells [7].

Myeloid‐derived suppressor cells (MDSCs) are defined as a heterogenous group of different cellular subsets with myeloid origin that exhibit immune regulatory functions, characterized by their ability to inhibit T‐cell responses. MDSC are thought to play an important role in the tumor microenvironment, where they suppress anti‐tumor responses and promote tumor progression. Reactive oxygen species (ROS), arginase‐1, and nitric oxide, are a few of the molecules that have been suggested to mediate immune suppression by MDSC [8].

MDSC can be divided into three different subsets based on their morphological and phenotypic features. The two main groups are the polymorphonuclear (PMN)‐MDSC and the monocytic (M)‐MDSC, but a third subset called early‐stage (e)MDSC has also been suggested in humans [8–10]. The PMN‐MDSC are neutrophil‐like cells, while the M‐MDSC are monocyte‐like cells [8]. The different subsets have unique, but partly overlapping, functional characteristics as well as biochemical traits [8]. Immaturity is a common trait in MDSC, however, the eMDSC have a more immature origin compared to the PMN‐MDSC [8].

Studying MDSC in humans is difficult, since they are present in low numbers [11], hence it is difficult to perform functional assays and evaluate their precise phenotype and function [11]. Another issue is that MDSC lack specific cell surface markers that can distinguish them from other cell types [11]. PMN‐MDSC are categorized as CD11b^+^CD14^−^CD15^+^ or CD11b^+^CD14^−^CD66^+^ cells, while M‐MDSC are categorized as CD11b^+^CD14^+^HLA‐DR^−/low^CD15^−^ cells [8]. These markers are also present on mature neutrophils and monocytes, respectively. The eMDSC subset is characterized as Lin^−^HLA‐DR^−^CD33^+^, where Lin^−^ (lineage negative) includes CD3, CD14, CD15, CD19, CD20, and CD56 [8]. To distinguish PMN‐MDSCs from mature neutrophils, density gradient centrifugation is needed [8]. PMN‐MDSCs accumulate in the low‐density fraction alongside mononuclear cells and are consequently termed low‐density granulocytes (LDGs) in autoimmune disease contexts. Mature neutrophils accumulate in high‐density layer with other granulocytes and red blood cells. These neutrophils are often referred to as normal density granulocytes or mature neutrophils, which is used in this manuscript.

The class E scavenger receptor lectin‐type oxidized LDL receptor 1 (LOX‐1) has been suggested to be a PMN‐MDSC specific marker [12]. LOX‐1 is mainly expressed on other cell types, for example, macrophages and endothelial cells, and seems to be increased in different types of cancer where it is associated with cancer progression and metastasis formation [12, 13]. However, it is uncertain if LOX‐1 could be used as a specific PMN‐MDSC marker, since others have shown that it is expressed on mature neutrophils and not specific for patient PMN‐MDSC [14, 15].

Increased levels of MDSC have been observed in different types of cancer and are often correlated with poor prognosis [11]. MDSC are thought to be induced by the tumor microenvironment where they promote a pro‐tumor environment by altering the immune response, leading to tumor progression [16].

In MM, an increase of PMN‐MDSC in the blood has been reported to correlate with disease progression and promote resistance to therapy [17–19]. PMN‐MDSC have also been found in the BM, where they inhibit T‐cell functions via ROS and Arginase 1 production [18, 20]. In addition, BM derived PMN‐MDSC increased the proliferation of MM plasma cells in vitro [20]. PMN‐MDSC does, however, not seem to be increased in MGUS patients [21]. Condamine et al. [12] have seen an increase of LOX‐1 on PMN‐MDSC in the BM of MM patients, compared to in the blood.

Some suggest that M‐MDSC levels are increased in MM patients, while others claim the opposite [17]. There have also been suggestions that M‐MDSC from MM patients induce T‐reg formation, and that they increase in the blood after treatment with lenalidomide [22–24]. Many of the papers describing MDSC levels do not consider their suppressive ability, since it is difficult to obtain a large enough quantity of the cells to perform the necessary experiments.

When it comes to MDSC in general, the data has been elusive. Some suggest increased levels of certain MDSC subsets, while others claim the opposite, studying the same diseases. To our knowledge, eMDSC levels have not been described in neither MM, nor MGUS.

In this study we want to evaluate the immune regulatory role of MDSC in MGUS and MM, by investigating the levels and inhibitory function of M‐MDSC, PMN‐MDSC, and the rarely investigated subset of eMDSC, in both bone marrow and blood. We will also investigate if LOX‐1 can be used as a specific PMN‐MDSC marker and compare their suppressive ability to mature neutrophils.

## 2. Materials and Methods

### 2.1. Patients and Controls

Patients with a newly discovered M‐spike or symptoms consistent with plasma cell disorders, referred to the Department of Hematology, Skåne University Hospital, Lund, were included during the period 2018–2021. The patients included in the study were recently diagnosed with MGUS or MM and had not received treatment for the disease. Patient characteristics can be viewed in Table S1. All patients were diagnosed according to the international myeloma working group criteria. None of the patients had any ongoing symptoms of infection. BM and peripheral blood were collected from MGUS patients (*n* = 7), MM patients (*n* = 11), and healthy controls (*n* = 12). The study was approved by the regional ethical review board in Lund, Sweden, ref no 2016/768. Samples were taken after the participants, both patients and healthy controls, had signed written informed consents.

### 2.2. Sample Preparation

Each peripheral blood and bone marrow aspirate was separated by Lymphoprep (Stemcell technologies, Cambridge, UK) density gradient centrifugation. The mononuclear layer was used for further MDSC and lymphocyte separation/analysis, and the polynuclear cell/erythrocyte layer was used for mature neutrophil separation. Cells were typically processed and analyzed on a per‐donor basis, with one patient or healthy donor handled each day. The number of independent experimental replicates is indicated in the figure legends.

### 2.3. Isolation of MDSC Subsets

The isolated mononuclear cells were stained with a MDSC antibody panel for flow cytometry, see Table S3 and Figure S1 for gating strategy. The samples were put on ice after staining and the PMN‐MDSC, M‐MDSC, and eMDSC were sorted using the fluorescence activated cell sorting (FACS) Aria Fusion (BD, Franklin Lakes, NJ, United States). The gating strategy can be viewed in Figure S1. PMN‐MDSC were defined as CD45^+^Lin^−^CD33^+/dim^HLA‐DR^−^CD66b^+^CD11b^+^, while M‐MDSC were defined as CD14^+^HLA‐DR^−/low^ and eMDSC as CD45^+^Lin^−^CD33^+/dim^HLA‐DR^−^CD66b^−^CD11b^+^. The cells were sorted into cell culture medium: RPMI‐1640 without L‐glutamine (Sigma, Malmö, Sweden) supplemented with 10 % fetal calf serum (Gibco, Thermo Fisher, Waltham, MA, United States) 10^4^ U/mL penicillin (Gibco, Thermo Fisher), 10 ng/mL streptomycin (Gibco, Thermo Fisher) and 2 mM L‐glutamine (Gibco, Thermo Fisher). The MDSC were used in the T‐cell proliferation assay immediately after sorting.

### 2.4. Isolation of Neutrophils

The isolated polynuclear cells were treated with 0.84% NH_4_Cl to lyse the erythrocytes included in this layer. The mature neutrophils were further isolated using an EasySep human neutrophil isolation kit (Stemcell Technologies, Cambridge, UK) according to manufacturer’s instructions. The purity was checked by flow cytometry and was > 95%. Impurities did not form any clear cell populations and was considered debris mixed with occasional lymphocyte or monocyte. In one control experiment, mature neutrophils were isolated from the lysed RBC layer using FACS. Gimsa staining was used to confirm mature neutrophils in some experiments.

### 2.5. T‐Cell Proliferation Assay

The T‐cell proliferation assay was performed as described previously [25, 26]. Briefly, 100,000 CFSE labeled healthy donor T‐cells were co‐cultured with 50,000 FACS sorted MDSC—either M‐MDSC, PMN‐MDSC, or eMDSC. After 3 days, the proliferation of the T‐cells was measured on a CytoFLEX (Beckman Coulter).

In some experiments the PMN‐MDSCs and eMDSCs were preactivated by adding 1 *μ*M of the neutrophil activator N‐formylmethionyl‐leucyl‐phenylalanine (fMLF). To evaluate the role of ROS production, the ROS inhibitor catalase (Sigma, Malmö, Sweden) was added to some experiments as indicated.

### 2.6. Statistics

Statistical analysis was performed using GraphPad Prism Version 9 (GraphPad Software, San Diego, CA, United States). Due to a low sample size, the data was assumed to be non‐normally distributed and nonparametric tests were used. For groups > 2, the Kruskal–Wallis test was used to compare groups. Dunn’s multiple comparison test was used for multiple comparisons. When comparing two groups, the Mann–Whitney test was used. We assumed the difference in proliferation to be normally distributed. In groups with more than four experiments, paired *t*‐test was used to evaluate significance.

## 3. Results

### 3.1. eMDSC Are Decreased in the Blood of MM Patients

Since MM is a disease of the BM, we wanted to investigate the levels of all MDSC subsets in BM. As blood is more accessible and more commonly evaluated, we wanted to compare the levels of BM MDSC to the levels found in blood.

In the BM, there were no statistically significant differences in the levels of PMN‐MDSC, M‐MDSC, or eMDSC between the healthy donors, MGUS patients, and MM patients (Figure 1a–c). There were also no statistically significant differences of PMN‐MDSC or M‐MDSC in the blood (Figure 1d,e). Interestingly, eMDSC is decreased in the blood of MM patients, compared to healthy donors (*p* = 0.041) (Figure 1f).

Figure 1Levels of PMN‐MDSC, M‐MDSC, and eMDSC in the BM (a–c) and blood (d–f) of healthy donors, MGUS patients and MM patients. The levels are calculated as the percentage of single cells. Bars indicate median with 95% CI and statistical significance was tested using Kruskal–Wallis test and Dunn’s multiple comparison test ( ^∗^ *p* < 0.05). Bone marrow; HD (*n* = 11), MGUS (*n* = 7), and MM (*n* = 11). Blood; HD (*n* = 10), MGUS (*n* = 5), and MM (*n* = 11). Summary of 24 independent experiments.

**Figure:**
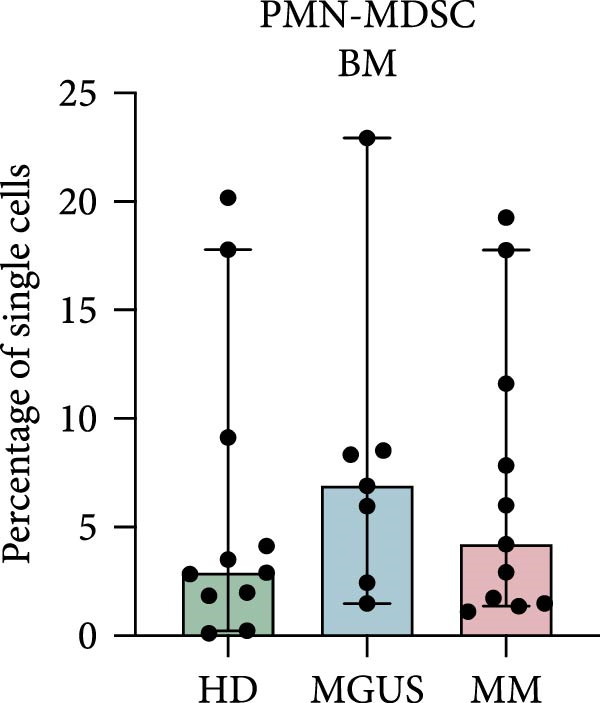
(a)

**Figure:**
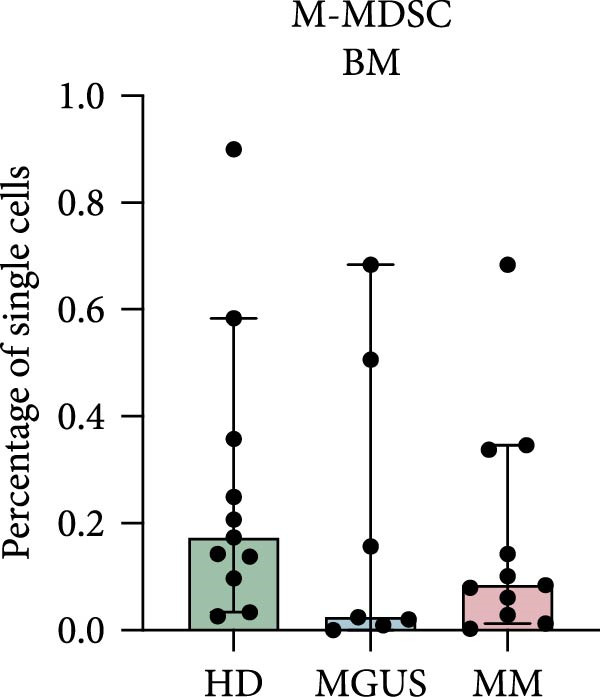
(b)

**Figure:**
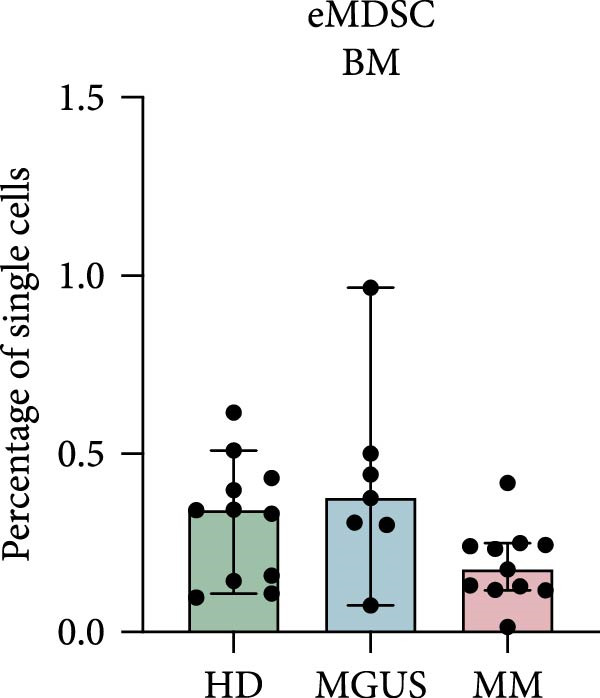
(c)

**Figure:**
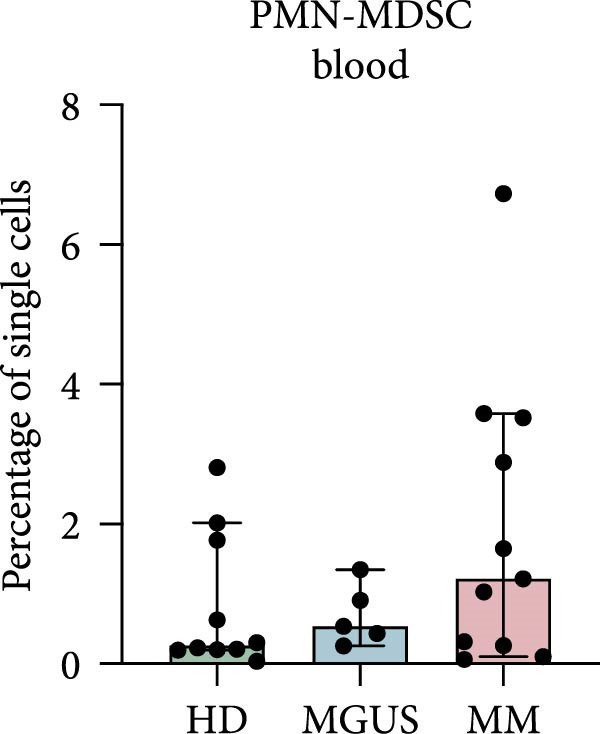
(d)

**Figure:**
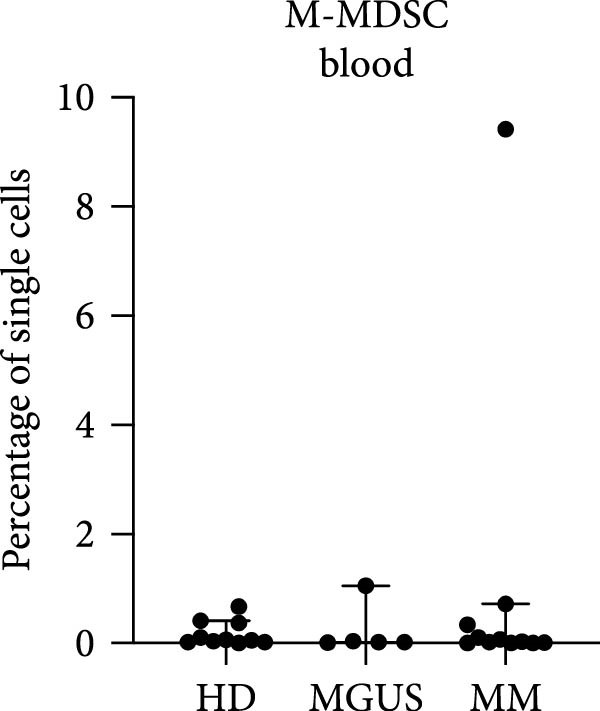
(e)

**Figure:**
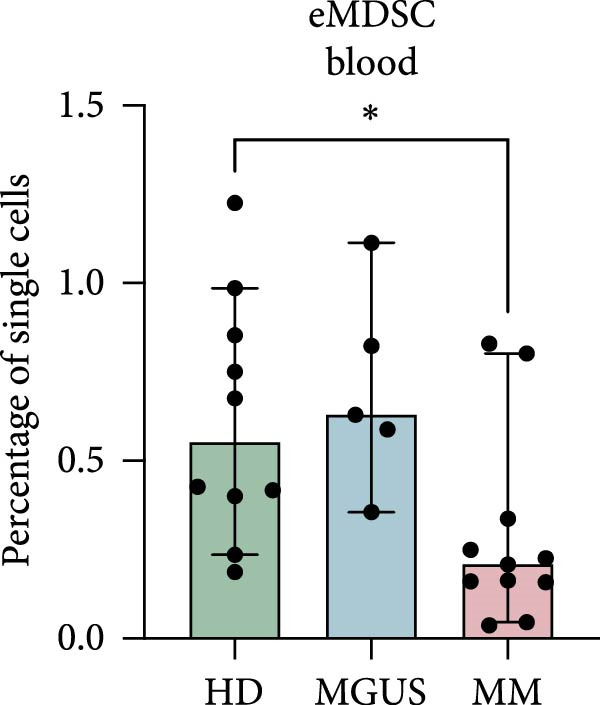
(f)

### 3.2. LOX‐1 Is Not a Specific Marker for Patient PMN‐MDSC

LOX‐1 has been suggested to be a specific PMN‐MDSC marker, only expressed in high levels on PMN‐MDSC from cancer patients [12]. It has also been suggested that MM patients have increased expression of LOX‐1 in the BM, compared to in the blood [12]. Therefore, we wanted to investigate the levels of LOX‐1 and determine if it could be used as a specific PMN‐MDSC marker in MM.

Indeed, BM PMN‐MDSC from MM patients have a higher expression of LOX‐1 compared to blood PMN‐MDSC from the same patient (*p* = 0.0004) (Figure 2c). However, PMN‐MDSC from HD and MGUS patients also express LOX‐1 and at similar levels as PMN‐MDSC from MM patients (Figure 2a,b). These data indicate that LOX‐1 is not a specific PMN‐MDSC marker, only found on cancer patients. In addition, LOX‐1 expression levels seem not to be increased in the BM of MM patients and are present also in a healthy setting.

Figure 2LOX‐1 expression in PMN‐MDSC. (a) The geometric mean of LOX‐1 in PMN‐MDSC in the BM of healthy donors (*n* = 11), MGUS patients (*n* = 6), and MM patients (*n* = 10). (b) The geometric mean of LOX‐1 in PMN‐MDSC from the blood of healthy donors (*n* = 10), MGUS patients (*n* = 5), and MM patients (*n* = 11). (c) Comparison of the geometric mean of LOX‐1 in PMN‐MDSC from the blood and the bone marrow of MM patients. Statistical significance for (a) and (b) was tested using Kruskal–Wallis test and Mann–Whitney was used to test the significance in (c) ( ^∗∗∗^ *p* < 0.001). Bars indicate median with 95% CI. Summary of 24 independent experiments.

**Figure:**
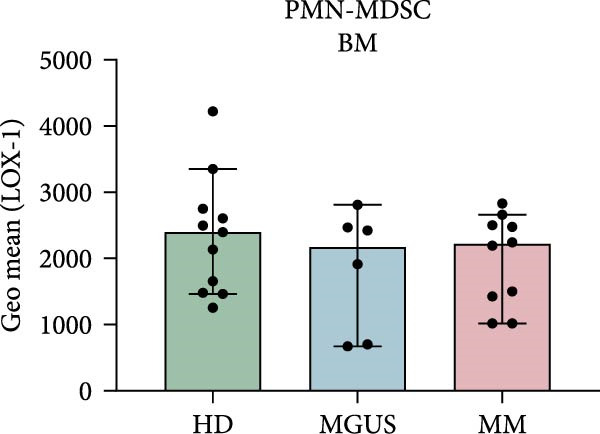
(a)

**Figure:**
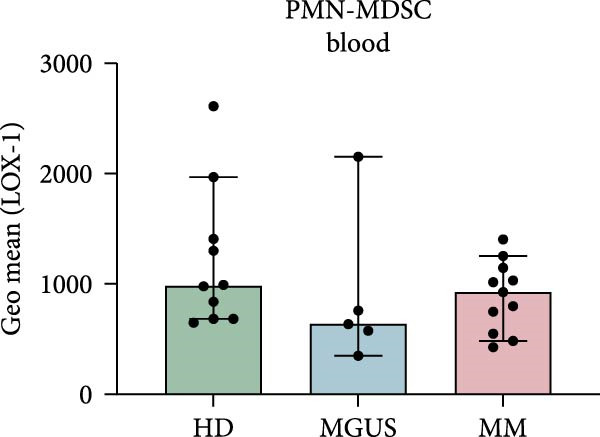
(b)

**Figure:**
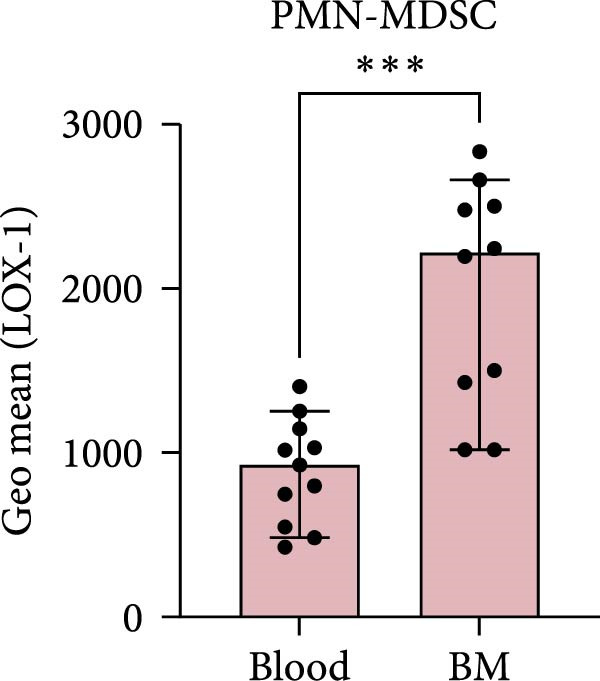
(c)

### 3.3. The Isolated MDSC Subsets Are Not Strong Inhibitors of T‐Cell Proliferation

The main characteristic for all MDSC subtypes is their ability to suppress T‐cell responses, including T‐cell proliferation. The ability of BM PMN‐MDSC to inhibit T‐cell proliferation showed a great variability and cells from some individuals inhibit proliferation, while others increased proliferation (Figure 3a). No trend towards differences in inhibitory effect was found between the different groups. However, PMN‐MDSCs from the blood of healthy donors, MGUS patients, and MM patients all had a small inhibitory effect (Figure 3b). However, the numbers of successful experiments and level of inhibition are too low to reach significance.

Figure 3The effect of PMN‐MDSC on T‐cell proliferation. (a) BM PMN‐MDSC from healthy donors (*n* = 5), MGUS patients (*n* = 3), and MM patients (*n* = 5) and their effect on T‐cell proliferation. (b) PMN‐MDSC from the blood of healthy donors (*n* = 3), MGUS patients (*n* = 1), and MM patients (*n* = 4) and how they inhibit T‐cell proliferation. Bars indicate median with 95% CI. For >2 group comparison, statistical significance was tested with Kruskal–Wallis test, and for two group comparison paired *t*‐test was used. Summary of 10 independent experiments.

**Figure:**
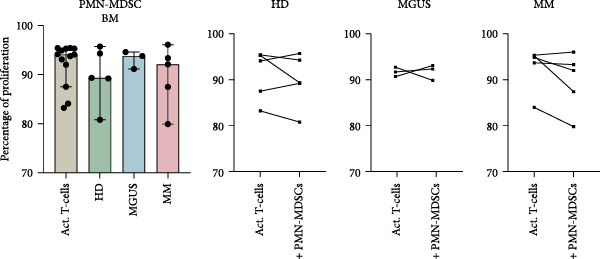
(a)

**Figure:**
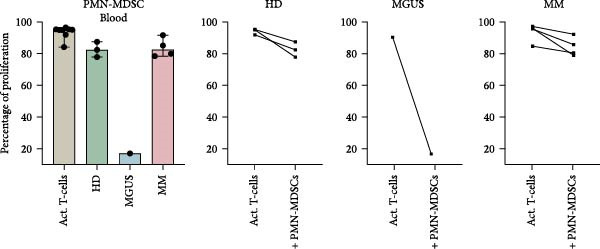
(b)

BM M‐MDSC did not inhibit T‐cell proliferation, instead a minor increase of the proliferation with a few percentile was seen in most experiments (Figure 4a). In BM M‐MDSC from healthy donors, the trend reached significance (*p* = 0.02). Blood M‐MDSC shows a similar trend as BM M‐MDSC (Figure 4b). BM eMDSC did not affect the T cell proliferation (Figure 5), nor did blood eMDSC (Figure 5b). In summary none of the MDSC populations had a strong inhibitory effect on T cell proliferation.

Figure 4The effect of M‐MDSC on T‐cell proliferation. (a) BM M‐MDSC from healthy donors (*n* = 4), MGUS patients (*n* = 3), and MM patients (*n* = 3) and their effect on T‐cell proliferation. (b) M‐MDSC from the blood of healthy donors (*n* = 1), MGUS patients (*n* = 1), and MM patients (*n* = 1) and their effect on T‐cell proliferation. Bars indicate median with 95% CI. For >2 group comparison, statistical significance was tested with Kruskal–Wallis test, and for two group comparison paired *t*‐test was used. Summary of 10 independent experiments.  ^∗^ *p* < 0.05.

**Figure:**
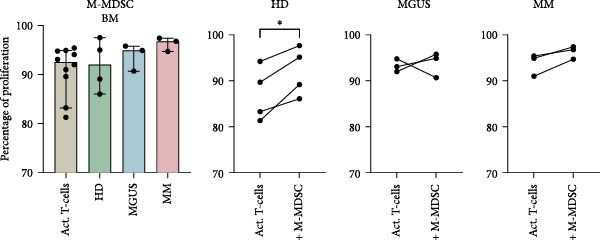
(a)

**Figure:**
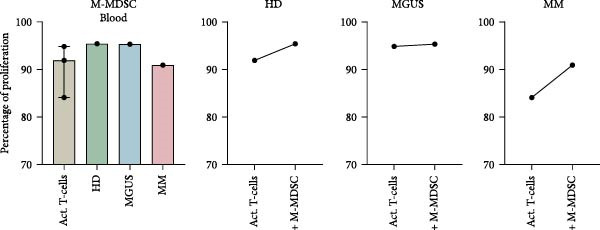
(b)

Figure 5The effect of eMDSC on T‐cell proliferation. (a) BM eMDSC from healthy donors (HD) (*n* = 4), MGUS patients (*n* = 3), and MM patients (*n* = 1) and their effect on T‐cell proliferation. (b) eMDSC from the blood of healthy donors (*n* = 2), MGUS patients (*n* = 1), and MM patients (*n* = 2) and their effect on T‐cell proliferation. Bars indicate median with 95% CI. For > 2 group comparison, statistical significance was tested with Kruskal–Wallis test, and for two group comparison paired *t*‐test was used. Summary of 10 independent experiments.

**Figure:**
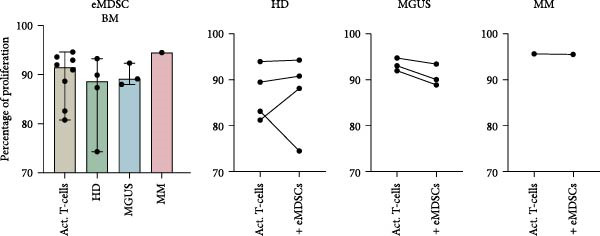
(a)

**Figure:**
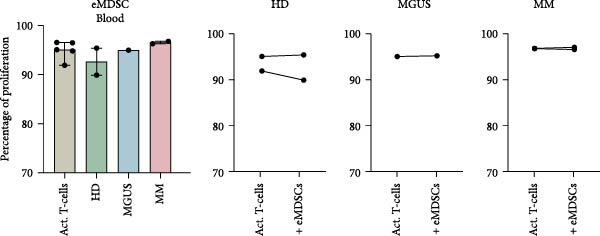
(b)

### 3.4. PMN‐MDSC Are Not as Suppressive as Mature Neutrophils in T‐Cell Proliferations Assays

As PMN‐MDSC and mature neutrophils are thought to be very similar, we compared their ability to suppress T‐cell proliferation. Previously published data from our group describe that mature neutrophils can suppress T‐cell responses in a similar manner as MDSC [25].

In this study, we found that PMN‐MDSC are not as suppressive as mature neutrophils (Figure 6). Blood mature neutrophils from both healthy donors (HD; *p*  < 0.0001) and MM patients (*p*  < 0.0001) strongly inhibited T‐cell proliferation, whereas blood PMN‐MDSCs exhibited only a marginal suppressive effect (Figures 3b and 6a). When comparing the suppressive effect of blood PMN‐MDSC with mature neutrophils from the same MM patients (*n* = 4), mature neutrophils were more suppressive than PMN‐MDSC (*p* = 0.038) (Figure 6b). In the BM, only MM mature neutrophils had a major suppressive effect (*p* = 0.0006) (Figure 6b). MM PMN‐MDSC, healthy donor mature neutrophils and healthy donor PMN‐MDSC were less suppressive. In all cases but one, the PMN‐MDSC were less suppressive than mature neutrophils from the same donor (*n* = 5). Taken together, these data indicate that PMN‐MDSC are less T‐cell suppressive than mature neutrophils.

Figure 6The inhibitory effect of PMN‐MDSC versus mature neutrophils in the blood and BM. (a) T‐cell proliferation in the presence of healthy donor mature neutrophils (*n* = 9), healthy donor PMN‐MDSC (*n* = 3), MM patient mature neutrophils (*n* = 8), and MM PMN‐MDSC (*n* = 5) isolated from the blood. (b) Individual comparison of inhibitory effect between blood PMN‐MDSC and mature granulocytes from the same MM patient (*n* = 4). (c) T‐cell proliferation in the presence of healthy donor mature neutrophils (*n* = 8), healthy donor PMN‐MDSC (*n* = 5), MM patient mature neutrophils (*n* = 8), and MM PMN‐MDSC (*n* = 5) isolated from the bone marrow. (d) Individual comparison of inhibitory effect between bone marrow PMN‐MDSC and mature granulocytes for the same MM patient (*n* = 5). Bars indicate median with 95% CI and statistical significance was tested using Kruskal–Wallis test and Dunn’s multiple comparison test. For two group comparison paired *t*‐test was used. Summary of 20 independent experiments.  ^∗^ *p* < 0.05,  ^∗∗∗^ *p* < 0.001, and  ^∗∗∗∗^ *p* < 0.0001.

**Figure:**
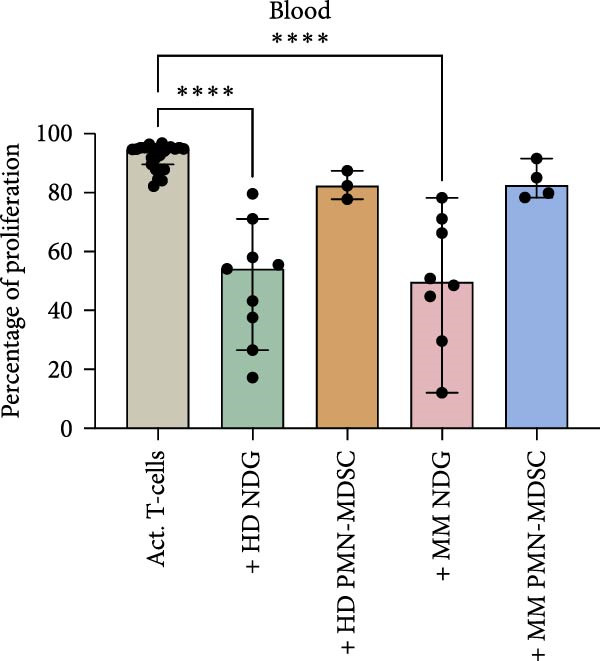
(a)

**Figure:**
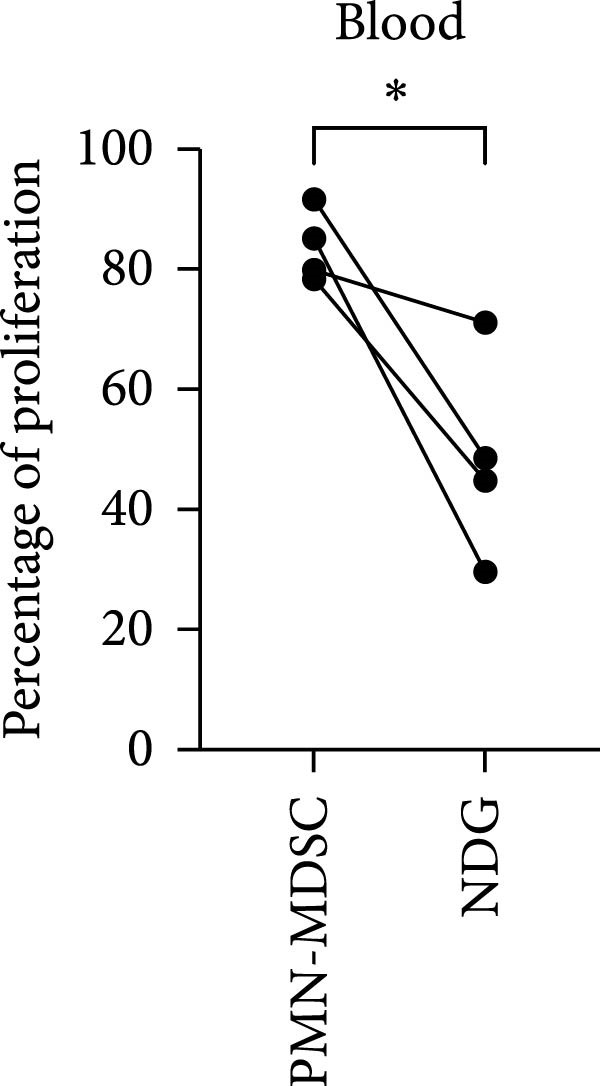
(b)

**Figure:**
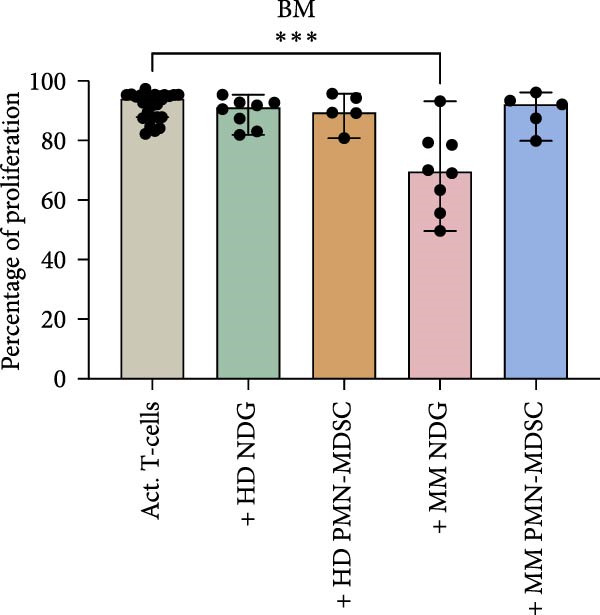
(c)

**Figure:**
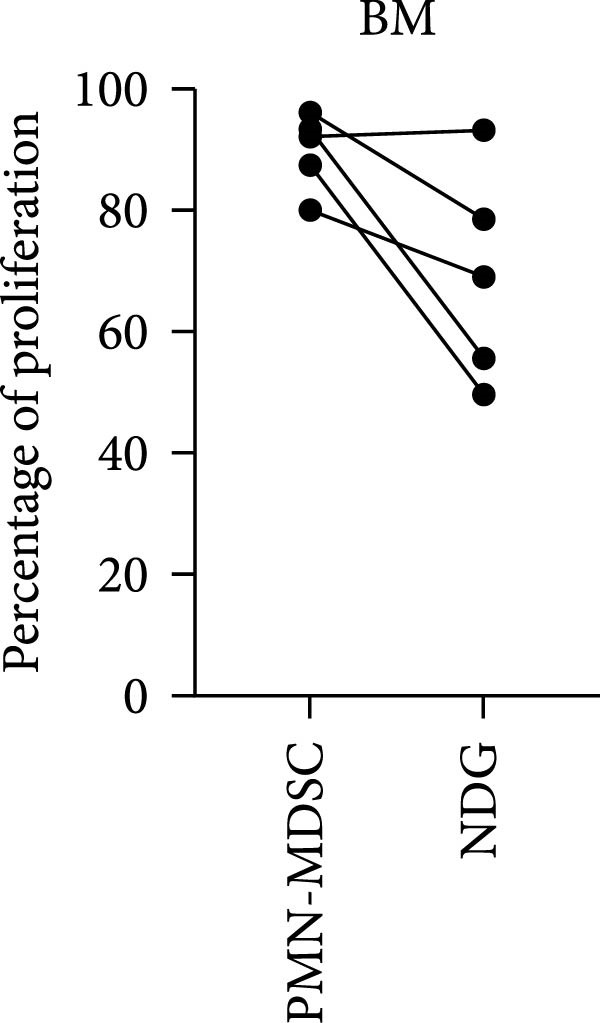
(d)

The observed difference in inhibition by PMN‐MDSC and mature neutrophils could be caused by different isolation methods. PMN‐MDSC are isolated using FACS, while mature neutrophils are isolated by magnetic separation. To test if the isolation process affected the inhibitory ability, we isolated mature neutrophils using the two different methods and compared them in the T‐cell proliferation assay. We found that FACS sorting did not affect the mature neutrophils’ ability to suppress T‐cell proliferation (Figure S2). Since PMN‐MDSC and mature neutrophils are supposed to be very similar, we conclude that FACS sorting does not affect the PMN‐MDSC ability to suppress T‐cells.

### 3.5. Activation of Blood PMN‐MDSC Increase Suppression of T‐Cell Proliferation

Since we and others have shown that neutrophil activator fMLF can enhance mature neutrophils suppression on T‐cell proliferation. We also wanted to investigate if fMLF activation of PMN‐MDSC could increase their suppressive ability. fMLF only had a minor effect on PMN‐MDSC from the BM, but a more pronounced effect on PMN‐MDSC from the blood (Figure 7). Indicating that the suppressive effect of blood PMN‐MDSC can be increased by fMLF activation.

Figure 7The effect of fMLF activated PMN‐MDSC on T‐cell proliferation in (a) bone marrow and (b) blood in one representative experiment.

**Figure:**
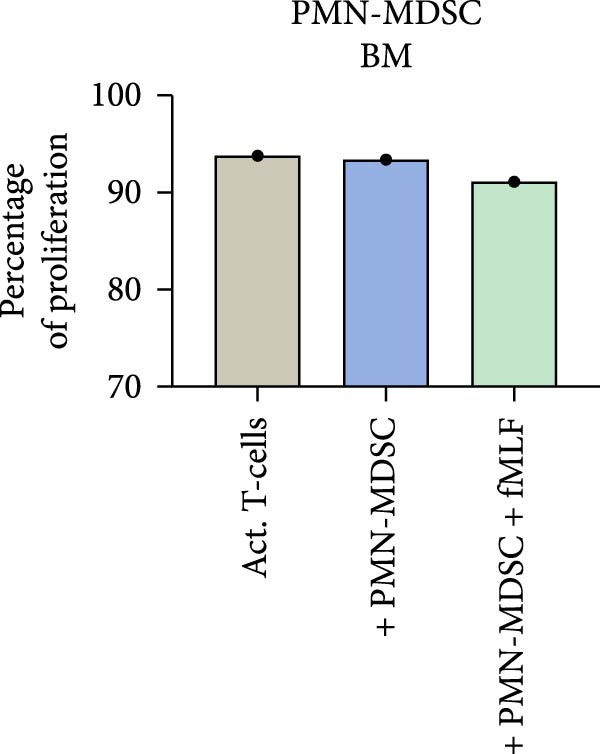
(a)

**Figure:**
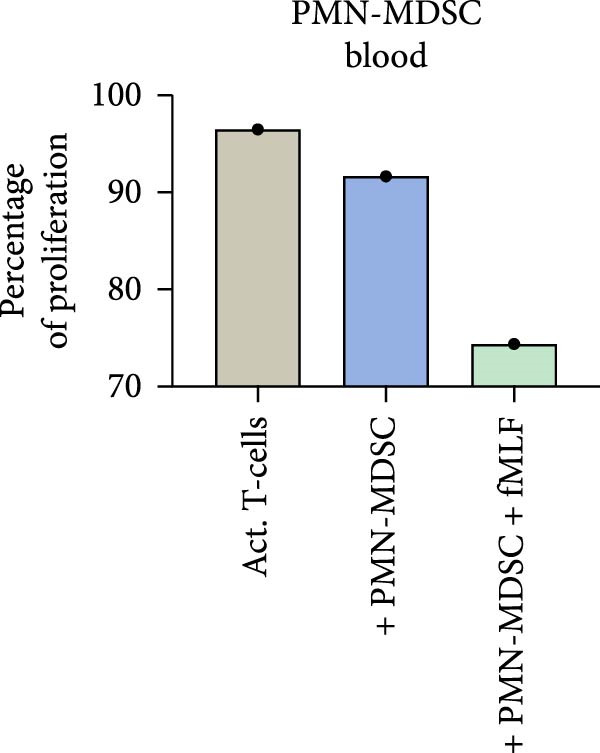
(b)

### 3.6. PMN‐MDSC Suppression of T‐Cell Proliferation are Mediated by ROS

ROS are important in mature neutrophils mediated T‐cell inhibition. To test whether PMN‐MDSC suppression is mediated by ROS, the ROS inhibitor catalase was added to the co‐cultures to counteract the suppressive effect. Catalase abrogated the suppression of T‐cell proliferation in bone marrow and in blood in MM patients, MGUS patients, and healthy donors (Figure 8 and not shown). These data indicate that the suppressive effect of PMN‐MDSC may be caused by ROS production.

Figure 8The ROS inhibitor catalase abrogates the suppressive effect of PMN‐MDSC from (a) bone marrow and (b) blood on T‐cell proliferation. It is based on two independent experiments.

**Figure:**
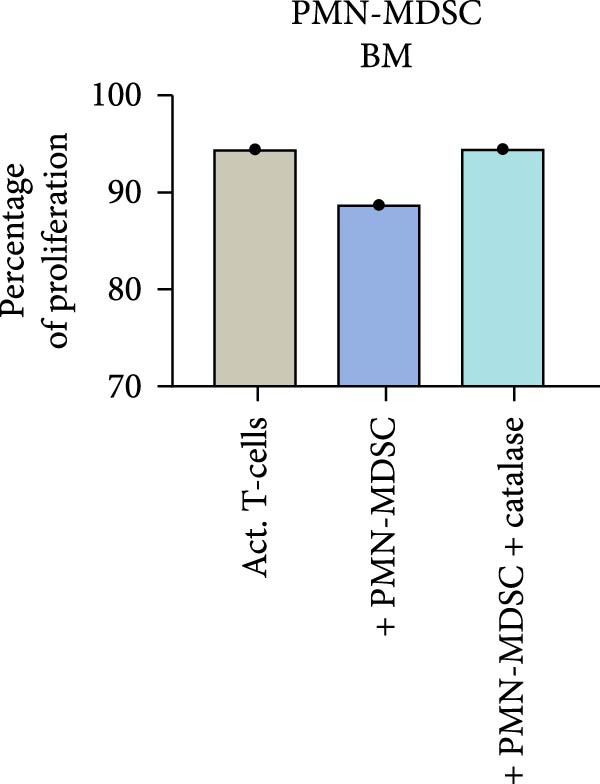
(a)

**Figure:**
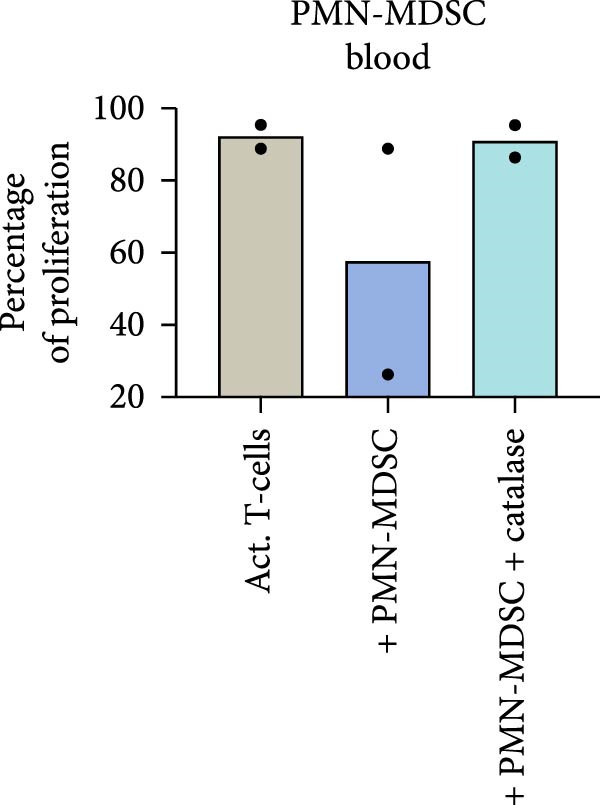
(b)

## 4. Discussion

MDSC are thought to play an important role in the tumor microenvironment, where they through different molecules and cytokines can promote tumor immune evasion using several different mechanisms. In this study we provide insight into how the different MDSC subsets act in the bone marrow and blood of MGUS and MM patients.

We did not find increased numbers of PMN‐MDSC in bone marrow or blood of MGUS and MM patients. Other researchers have observed increased levels of PMN‐MDSC in the BM [17, 27] and blood of MM patients [17, 19, 20, 23, 28]. We have only investigated samples from newly diagnosed, untreated patients. Other studies have focused on other MM patient groups, such as relapsed MM patients. In MM patients, PMN‐MDSC have been shown to increase with disease progression [20] and correlate with a poor prognosis [27]. Like our results, newly diagnosed patients did not have an increase of PMN‐MDSC in their blood [20]. Blood‐derived PMN‐MDSCs show modest suppression of T‐cell proliferation, though their inhibitory effect is weaker than that of mature neutrophils. Notably, this suppressive activity is consistent across healthy donors, MGUS patients, and MM patients, suggesting disease status does not significantly influence PMN‐MDSC function. This is in line with data from Favaloro et al. [19], that blood PMN‐MDSCs from MM patients are as suppressive as from healthy donors. However, others show contradictory results; that MDSC from MM patients are highly suppressive, while healthy donor MDSC lacks this ability [17]. T‐cell proliferation suppression could be increased by activating PMN‐MDSC with neutrophil activator fMLF and seems to be mediated by ROS as the radical scavenger catalase abrogates the inhibitor effect.

In 2010, M‐MDSC was described to be increased in the blood of newly diagnosed MM patients [22]. This is not in line with the results in this study, furthermore, Ramchandran et al. [17] have, like us, reported that M‐MDSC levels are not increased in MM patients. However, their data indicate that M‐MDSC from MM patients can inhibit T‐cell responses, a finding that does not agree with our data. Studies have shown that normal monocytes can increase T‐cell proliferation [29], and in our hands the M‐MDSC act in a similar way. The gating of HLA‐DR^−/low^ cells is complex, since there is no clear limit between HLA‐DR^+^ cells, and HLA‐DR^−/low^ cells. It is possible that different gating strategies yield different results and that our population has contained monocytes as M‐MDSC seem to be lacking. It is also possible that the patients in other studies have had a more severe disease compared to the patients in this study, as it has been claimed that M‐MDSC levels are higher in patients with progressive disease and correlate with a poor prognosis and low overall survival [30].

To our knowledge, no other researchers have evaluated the level and function of eMDSC in the blood and BM of MGUS or MM patients compared to healthy donor. Interestingly, we found a decrease of eMDSC in the blood of MM patients, compared to healthy donors. Casetta et al. [14] has investigated eMDSC levels in six different solid cancers and saw that in 5/6 cancer types the levels of eMDSC in the blood was the same as in healthy donors. However, like us, in 1/6 cancer types the eMDSC level was decreased in the cancer patients. We did, unlike them, not observe a T‐cell suppressive response [14].

In recent years it has become evident that neutrophils also exhibit immune regulatory functions, in the same manner as PMN‐MDSC [15, 25, 26, 31–36]. Previous data from our group indicate that blood mature neutrophils, from both MM patients and healthy individuals, can suppress T‐cell proliferation and IFN‐*γ* production [25]. In this study, we compared the suppressive ability of PMN‐MDSC to mature neutrophils and found that mature neutrophils are stronger T‐cell suppressors than PMN‐MDSC. Others have done similar experiments and found limited differences [36]. Since the major difference between PMN‐MDSC and mature neutrophils seem to be their density it is possible that PMN‐MDSC are ordinary neutrophils with less granules, as granularity is directly proportional to density [37]. PMN‐MDSC are suggested to be more immature than mature neutrophils, and during maturation, neutrophils increase in both size and granularity [37].

One of the major problems with MDSC research, is that there has been no consensus on which markers to use for their identification, and the fact that the different subsets lack specific markers. Two papers have been published, where the authors have set up a few guidelines for the identification of PMN‐MDSC, M‐MDSC, and eMDSC in blood [8, 14]. Without these guidelines it is difficult to compare the results from different groups, as it is not certain that the same cells are investigated. LOX‐1, a class E scavenger receptor, has been suggested to be a specific PMN‐MDSC marker [12]. However, when others have tried to reproduce these findings, it has failed [14, 15]. LOX‐1 seems to be expressed not only on PMN‐MDSC, but also on mature neutrophils, making it a poor marker for PMN‐MDSC [15]. It has also been suggested that PMN‐MDSC from the BM of MM patients have a higher expression of LOX‐1 than PMN‐MDSC from the blood of the same patient [12]. We and others have shown that LOX‐1 is present on PMN‐MDSC, but that the expression levels do not differ from the PMN‐MDSC found in healthy donors [14]. Several different methods are used to functionally test suppression of T‐cell proliferation, complicating the comparison between studies such as lipopolysaccharide matured dendritic cells to activate the T‐cells [17], CD3/CD28 coated beads [19] or CD3/CD28 coated plates [14], or the use of PBMCs instead of pure T‐cells. Another limitation in MDSC research is due to their low numbers. It is difficult to obtain enough cells for experiments in all patients, which could create a bias and individuals with high amounts of MDSC are more likely to be evaluated.

In a recent study using unbiased single‐cell RNA sequencing, Rivera et al. [38] has delineated three dominant CXCR2‐expressing neutrophil subpopulations within the MM microenvironment. These subsets are defined by distinct transcriptional signatures: TREM1^+^CD10^+^, RETN^+^LCN2^+^, and TNFAIP3^+^CXCL8^+^. Functionally, the CXCR2^+^CD10^+^ subset was identified as possessing T‐cell suppressive capabilities, suggesting a pro‐tumorigenic role. The study further indicates that MM tumor cells recruit and activate these neutrophils primarily via the CXCL8–CXCR2 axis. This discovery of a defined immunosuppressive neutrophil subset justifies further investigation into its role in MM pathogenesis and its potential as a therapeutic target in MM and other malignancies.

Over the past decades, suppressive neutrophils have been described under various names—PMN‐MDSCs, granulocytic (G)‐MDSCs, low‐density neutrophils (LDNs), LDGs, and immature myeloid cells (IMCs). These terms all refer to neutrophil‐like cells isolated in the low‐density fraction after density gradient centrifugation, yet they are associated with different disease contexts. While these cells share a common isolation profile, their functional roles appear to vary across pathological conditions. This raises a critical question: do these designations represent distinct cell populations, such as the different maturation stages found by Rivera et al. [38] or are they simply reflections of neutrophil plasticity in response to diverse microenvironments?

## Conflicts of Interest

The authors declare no conflicts of interest.

## Author Contributions

Julia Westerlund, Thomas Hellmark, Åsa C. M. Johansson, and Markus Hansson contributed to the design of the study. Laboratory work was conducted by Julia Westerlund and Åsa Pettersson. Sandra Askman, Stina Wichert, and Markus Hansson recruited the patients. Julia Westerlund carried out the analysis, compiled the data, and drafted the manuscript. Julia Westerlund, Sandra Askman, Thomas Hellmark, Åsa C. M. Johansson, and Markus Hansson contributed to the writing of the final manuscript.

## Funding

The Swedish Cancer foundation, Wallenberg Centre for Molecular and Translational Medicine.

## Supporting Information

Additional supporting information can be found online in the Supporting Information section.

## Supporting information


**Supporting Information** Table S1. Patient characteristics. Table S2. Healthy donor characteristics. Table S3. MDSC Antibody panel for flow cytometry. Figure S1. Gating strategy for isolation of M‐MDSC, PMN‐MDSC, and eMDSC. Figure S2. FACS sorting do not affect the inhibitory effect of blood neutrophils.

## Data Availability

The data that support the findings of this study are available on request from the corresponding author. The data are not publicly available due to privacy or ethical restrictions.
