# Supplementary material for: Myeloid‐Derived Suppressor Cells (MDSCs) Suppress T‐Cell Proliferation Less Than Mature Neutrophils in Blood and Bone Marrow From Multiple Myeloma Patients
Source: J Immunol Res. 2026 Jan 5;2026:9232540. doi: 10.1155/jimr/9232540 (PMC12771638; doi:10.1155/jimr/9232540)
Supplement: Supplementary file 1 — Supporting Information Table S1. Patient characteristics. Table S2. Healthy donor characteristics. Table S3. MDSC Antibody panel for flow cytometry. Figure S1. Gating strategy for isolation of M‐MDSC, PMN‐MDSC, and eMDSC. Figure S2. FACS sorting do not affect the inhibitory effect of blood neutrophils. [file JIMR-2026-9232540-s001.docx]

**Supplementary materials**

Table 1. Patient characteristics

| Pat No | Sex | Age | Diagnosis | ISS | rISS | FISH | M component class | M protein (g/L) | % PCs BM |
| --- | --- | --- | --- | --- | --- | --- | --- | --- | --- |
| 1 | F | 80 | MGUS |  |  |  | IgG kappa | 12 | 5 |
| 2 | M | 84 | MGUS |  |  |  | IgA | 7 | 5 |
| 3 | F | 77 | MGUS |  |  |  | IgG kappa | 4 | 0.1 |
| 4 | M | 70 | MGUS |  |  |  | IgA lambda | 18 | 5 |
| 5 | F | 67 | MGUS |  |  |  | IgG kappa | 9 | 8 |
| 6 | M | 77 | MGUS |  |  |  | IgG lambda | 5 | 1 |
| 7 |  |  | MGUS |  |  |  |  |  |  |
| 8 | F | 64 | NDMM | II |  | None | IgG kappa | 45 | 16 |
| 9 | M | 46 | NDMM | I | II | Del(17p) | IgG lambda | 25 | 21 |
| 10 | M | 76 | NDMM | III | II | None | IgG lambda | 19 | 15 |
| 11 | M | 67 | NDMM | I | I | None | IgG kappa | 45 | 21 |
| 12 | F | 84 | NDMM | II | ND | ND | IgG lambda | 30 | 13 |
| 13 | M | 81 | NDMM | II |  | N/A | IgG kappa + IgM kappa | 18 | 4 |
| 14 | F | 72 | NDMM | II | II | None | IgD lambda + lambda | 21 | 35 |
| 15 |  | 45 | NDMM | I | II | Dup(1q) | IgA lambda | 20 | 12 |
| 16 | M | 74 | NDMM | II |  | Dup(1q) | IgA lambda | 12 | 24 |
| 17 | F | 51 | NDMM | ND | ND | Dup(1q) | IgG kappa | 40 | 11 |
| 18 | F | 79 | NDMM | II | II | None | IgG kappa | 23 | 15 |

NDMM = newly diagnosed multiple myeloma

ISS= International Staging System staging at diagnosis

rISS= revised ISS

FISH = Fluorescence in situ hybridization

%PCs BM=bone marrow plasmacells/myeloma cells at diagnosis

ND= Not Done

N/A= not available

Table 2. Healthy donor characteristics

| **Donor No** | **Sex** | **Age** |
| --- | --- | --- |
| 1 | N/A | N/A |
| 2 | N/A | N/A |
| 3 | N/A | N/A |
| 4 | M | 25 |
| 5 | F | 29 |
| 6 | F | 34 |
| 7 | F | 26 |
| 8 | M | 21 |
| 9 | F | 21 |
| 10 | F | 21 |
| 11 | M | 42 |
| 12 | N/A | N/A |

N/A = Not available

Table 3. MDSC Antibody panel for flow cytometry

| Antibody against | Fluorescent dye | Clone | Company |
| --- | --- | --- | --- |
| CD3 | APC-Cy7 | SK7 | BD Pharmingen |
| CD56 |  | HCD56 | BioLegend |
| CD19 |  | SJ25-C1 | BD Pharmingen |
| CD33 | APC | WM53 | BD Pharmingen |
| CD11b | V450 | ICRF44 | BD Horizon |
| CD15 | FITC | HI98 | BD Pharmingen |
| LOX-1 | PE | 15C4 | BioLegend |
| HLA-DR | PeCy7 | L243 | BD |
| CD66b | AF700 | G10F5 | BioLegend |
| CD14 | PerCP Cy5.5 | M5E2 | BD Pharmingen |
| CD45 | V500 | HI30 | BD Horizon |

Antibodies against CD3, CD56 and CD19 was used together as linage (Lin) marker, see supplemental figure 1 for gating strategy.

A B C

1

2

3

**Supplementary figure 1. Gating strategy for isolation of M-MDSC, PMN-MDSC and eMDSC.** The cells were gated from single cells. M-MDSCs were gated from monocytes (A1), based on CD14 and HLADR expression (A2). **M-MDSC** were defined as CD14^+^HLA-DR^-/low^(A2). To determine the limit for HLA-DR negativity a T-cell gate was used (not shown) and in some cases a fluorescence minus one (FMO) control for HLA-DR was used (not shown). **PMN-MDSC** were defined as CD45^+^Lin^-^CD33^+/dim^HLA-DR^-^CD66b^+^CD11b^+^, and can be gated using two different pathways (A1 to B3 or A1 to C3). **eMDSC** were defined as CD45^+^Lin^-^CD33^+/dim^HLA-DR^-^CD66b^-^CD11b^+^ (A1 to C3). Linage (Lin) includes CD3/CD56/CD19 antibodies.

Supplementary figure 2. FACS sorting do not affect the inhibitory effect of blood neutrophils.
